# Venous volume and compliance in the calf and forearm does not change after acute endurance exercise performed at continuous or interval workloads

**DOI:** 10.14814/phy2.15347

**Published:** 2022-06-07

**Authors:** Yasuhiro Iimura, Michiko Saito, Anna Oue

**Affiliations:** ^1^ Graduate School of Food and Nutritional Sciences Toyo University Gunma Japan; ^2^ Faculty of Food and Nutritional Sciences Toyo University Gunma Japan

**Keywords:** cycling exercise, single bout exercise, venous distensibility, venous occlusion plethysmography

## Abstract

Short‐term endurance exercise training for 6–8 weeks leads to increases in venous volume and compliance in the limbs. However, it is not known whether these venous vascular properties are improved by acute endurance exercise. We examined the effects of acute endurance exercise involving continuous or interval workloads on venous volume and compliance in the exercising (calf) and non‐exercising (forearm) limbs. Sixteen healthy young volunteers performed cycling exercise involving a continuous workload of 60% heart rate (HR) reserve or an interval workload of 40% HRreserve and 80% HRreserve, alternating every 2 min, for a total of 32 min each. Before and 60 min after acute cycling exercise, venous volume in the calf and forearm was measured by venous occlusion plethysmography during a cuff‐deflation protocol with a venous collecting cuff wrapped to the thigh and upper arm and strain gauges attached to the calf and forearm. The cuff pressure was maintained at 60 mmHg for 8 min and was then deflated to 0 mmHg at a rate of 1 mmHg/s. Venous compliance was calculated as the numerical derivative of the cuff pressure–limb venous volume curve. In both the calf and forearm, the cuff pressure–venous volume curve and the cuff pressure–venous compliance relationship did not differ between before and 60 min after exercise involving continuous or interval workloads. These results suggest that acute exercise does not improve venous volume and compliance in both the exercising and non‐exercising limbs.

## Graphical abstract

This result suggests that acute endurance exercise might not improve venous volume and compliance in both the exercising and non‐exercising limbs.

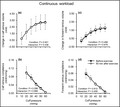

## 1 INTRODUCTION

Veins have high compliance and contain approximately two‐thirds of the total blood volume at rest (Greenfield & Patterson, 1956; Morris et al., 1974). When a person experiences physiological stress such as that during exercise, changes in venous volume, venous compliance, or both can cause a shift in blood volume from the veins to the heart. This shift in blood volume via the venous system plays an important role in circulatory regulation, including the maintenance of blood pressure (BP) and cardiac output (Rothe, 1983). In addition, the rise in venous stiffness seems to be involved, at least in part, in the elevation of BP and the pathogenesis of hypertension. Indeed, an association between lower venous compliance and higher BP has been reported in humans (Kooman et al., 1992; Oue et al., 2020; Safar & London, 1987; Takeshita & Mark, 1979) and animals (Fink et al., 2000; Overbeck, 1972; Simon, 1976). Therefore, from the viewpoint of preventing cardiovascular disease, it is important to ascertain what intervention might improve venous vascular health.

Endurance exercise is an established intervention for the improvement and maintenance of venous vascular health. For example, it has been reported that venous volume and compliance are increased in the exercising limb (Hernandez & Franke, 2004; Iida et al., 2011; Monahan et al., 2001; Oue et al., 2019) and that venous volume is increased in the non‐exercising limb (Miyachi et al., 2001; Oue et al., 2019) after endurance exercise training. In addition, the degree of such venous vascular adaptation with endurance exercise training is greater for interval workloads than for continuous workloads (Oue et al., 2019). However, the effect of acute endurance exercise on venous volume and compliance has not been investigated, and it is unclear whether these venous vascular properties increase in the exercising and non‐exercising limbs after acute endurance exercise or whether the degree of changes in these properties differs between interval and continuous workloads. Because exercise training consists of repeated bouts of acute endurance exercise, it is necessary to understand the effect of acute endurance exercise on venous vascular properties. It has been reported that the decrease in arterial stiffness after acute endurance exercise seems to be caused partly by enhanced endothelial function (Saz‐Lara et al., 2021; Trachsel et al., 2019). In addition, acute endurance exercise elicits the production of endothelial nitric oxide (NO) by increasing blood flow and shear stress (Mochizuki et al., 1999; Shen et al., 1995), and this enhanced NO synthesis modulates arterial stiffness (Wilkinson et al., 2002). In addition to the arterial side, the increased venous blood flow was found not only in the exercising but also in non‐exercising limbs during 30 min of cycling exercise (Ooue et al., 2008), and the production of NO resulting from stimulation of blood flow and shear stress was also observed in venous endothelial cells (Fukaya & Ohhashi, 1996; Noris et al., 1995). However, there is evidence that the number of endothelial cells, the amount of NO produced, and the sensitivity to NO are all lower in the veins than in the arteries (Burton, 1954; Fukaya & Ohhashi, 1996; Zhang et al., 2004; Zhang et al., 2006). From these findings, the expected improvement in venous endothelial function with acute endurance exercise would be negligible. Therefore, we hypothesized that venous volume and compliance in the exercising and non‐exercising limbs would not change after acute endurance exercise involving continuous or interval workloads.

Thus, to test our hypothesis, we investigated venous volume and compliance in the exercising (calf) and the non‐exercising (forearm) limbs before and after acute endurance exercise involving continuous or interval workloads.

## 2 MATERIALS AND METHODS

### 2.1 Participants

Sixteen young healthy adults (13 men and 3 women) participated in this study (age, 21.4 ± 1.7 years; height, 171.3 ± 9.2 cm; weight, 63.5 ± 7.2 kg). According to their medical histories and physical examinations, none of the participants had any chronic diseases. The participants abstained from caffeine, alcohol, and hard exercise for 24 h and from food intake for 2 h before the experiments. In addition, the female participants participated in the experiments during the follicular phase of their menstrual cycle (days 3–10 after the onset of menstruation) (Hernandez & Franke, 2004). The purpose, procedures, and risks of the study were explained to the participants and their informed consent was obtained. This study was approved by the Human Ethics Committee of the Toyo University and was conducted in accordance with the Declaration of Helsinki.

### 2.2 Experimental design

The participants came to our laboratory on four occasions, each separated by an interval of at least 24 h. On the first day, the participants practiced the cycling exercise. On the second day, they performed an incremental cycling exercise test to determine their workloads in the main experiments. On the third and fourth days, they participated in the main experiments.

### 2.3 Incremental cycling exercise test

Each participant performed an incremental cycling exercise test using a cycling ergometer (Ergometer 828 E, Monark Exercise AB, Vansbro, Sweden). The participants were asked to pedal on the cycle ergometer at a constant frequency of 60 rpm for 3 min at four different exercise intensities. Heart rate (HR) was monitored via an HR monitor (T31 coded transmitter; Polar Electro, Finland) and was recorded during the last 15 s of each step. To determine the workloads for the main experiment, the linear relationship between HR at the end of each step and the workloads was calculated, and then the workloads corresponding to HR at 40%, 60%, and 80% of HR reserve were estimated.

### 2.4 Main experiments

The main experiments were carried out twice in a room with the temperature maintained at 24.3 ± 0.3°C. After the participants rested in the supine position for at least 20 min, the change in calf and forearm venous volume was measured by venous occlusion plethysmography (VOP) during a cuff‐deflation protocol (Halliwill et al., 1999; Oue et al., 2019) in order to obtain pre‐exercise data. Following the recording of the baseline data, the participants performed a continuous cycling exercise or an interval cycling exercise for 32 min in the upright position, using the cycling ergometer. The continuous cycling exercise consisted of 60% HRreserve, while the interval cycling exercise consisted of repeating the 40% HRreserve and the 80% HRreserve every 2 min. Immediately after the 32 min of continuous or interval cycling exercises, the participants rested again in the supine position, and the change in calf and forearm venous volume was measured 60 min after the end of the exercise. Continuous and interval cycling exercises were performed randomly on separate days.

### 2.5 Assessment of venous vascular properties

The participants rested in the supine position with their left arm and leg raised above heart level. To measure the change in calf and forearm venous volume, the venous collecting cuff was wrapped around the left thigh and the left upper arm, and strain gauges were placed on the maximal thick site of the calf and forearm. Then, the collecting cuff was inflated to 60 mmHg for 8 min, after which the cuff pressure was manually reduced at a rate of 1 mmHg/s from 60 mmHg to 0 mmHg according to a previously described cuff deflation protocol (Halliwill et al., 1999; Oue et al., 2019). Throughout the cuff deflation protocol, changes in calf and forearm venous volume were measured noninvasively by VOP (EC4, D. E. Hokanson; Bellevue, WA). Because cuff inflation to 60 mmHg evokes fluid filtration, which can lead to overestimation of venous compliance, we corrected for the increase in venous volume caused by filtration, following the method in a previous study (Skoog et al., 2015).

Using the corrected venous volume in the calf and forearm, the relationship between cuff pressure and the change in calf and forearm venous volumes (i.e., the pressure–volume curve) was generated from the data points between 10 and 60 mmHg during the cuff‐deflation protocol. To avoid any a priori assumption regarding the cuff pressure (P)–venous volume (V) curve and to obtain the physiologic venous compliance relationship, venous compliance was calculated as the numerical derivative of each pair of cuff pressure–venous volume data points by the following equation (Oue et al., 2019).

$${\text{Venous compliance}}_{\mathrm{pi}}=\frac{\left(V_{i}-V_{i-10}\right)}{\left(P_{i}-P_{i-10}\right)}\;\left(\text{where}\;20\leq i\leq 60\right)$$

### 2.6 Measurements of BP, HR, and rating of perceived exertion

Systolic BP (SBP) and diastolic BP (DBP) were measured on the upper left arm with the auscultatory method using a sphygmomanometer (KM‐380; Kenzmedico, Saitama, Japan). Mean arterial BP (MAP) was calculated as follows: (SBP – DBP) / 3 + DBP. In addition, the rating of perceived exertion (RPE) was recorded using a Borg scale from 6 to 20 (Borg, 1970). During acute exercise, SBP, and DBP were measured every 8 min for both the continuous and interval cycling exercises, and HR was measured every 4 min for the continuous cycling exercise and every 2 min for the interval cycling exercise. In addition, RPE was measured every 4 min for both the continuous and interval workloads. While measuring the change in limb venous volume before and at 60 min after acute exercise, measurements of SBP, DBP, and HR were taken immediately after completion of the cuff‐deflation protocol.

### 2.7 Statistical analysis

Values are expressed as means ± standard deviation. To compare the time courses of HR, BP, and RPE during acute exercise at both continuous and interval workloads, one‐way analysis of variance (ANOVA) with repeated measures was performed to assess the effect of time. When a significant time effect was detected, the Bonferroni post hoc procedure was used to determine where differences occurred. To compare the changes in limb venous volume and venous compliance with the cuff pressure between before and after acute exercise, two‐way ANOVA with repeated measures was applied to the venous volume and venous compliance obtained with a cuff pressure of 10–60 mmHg before and at 60 min after acute exercise, using the cuff pressure and the condition (before and after exercise) as fixed factors. If the main effect of the condition and/or interaction was detected, a post hoc analysis was performed using a paired *t*‐test. A paired *t*‐test was used to compare HR and BP between before and 60 min after acute exercise. To test the lack of differences in the venous volume and compliance in calf and forearm before and after acute exercise, we performed the equivalence test in accordance with previous study (Matsumoto et al., 2019). When the 95% confidence interval (95%CI) for intergroup (before and after acute exercise) differences in each parameter (venous volume or compliance) was within the equivalent margin, we found that there was an equivalence. In present study, we defined the equivalent margins by averaging the data of standard deviations which reported previous studies (Oue et al., 2019; Oue et al., 2020). The equivalent margins of the venous volume in the calf and forearm at 60 mmHg of cuff pressure were ± 0.89 ml/dl and ± 0.72 ml/dl, and those of the venous compliance in the calf and forearm at 20 mmHg of cuff pressure were ± 0.030 ml/dl/mmHg and ± 0.022 ml/dl/mmHg, respectively. Statistical significance was accepted at *P* < 0.05 (SPSS 26.0 for Windows; IBM, Armonk, NY).

## 3 RESULTS

### 3.1 Changes in HR, BP, and RPE during exercise

HR increased gradually with continuous exercise, reaching 161 ± 12 bpm at 32 min of exercise. SBP and MAP also increased but DBP did not during continuous exercise; these values were 158 ± 18, 92 ± 11, and 59 ± 12 mmHg, respectively, at 32 min. RPE increased gradually with continuous exercise; the value was 14 ± 2 at 32 min.

HR as measured every 2 min during interval exercise varied according to the exercise intensity, reaching 149 ± 14 bpm at 30 min for 40%HRreserve and 170 ± 11 bpm at 32 min for 80%HRreserve. In addition, the average HR value for the last 4 min of interval exercise, which was included in 40%HRreserve and 80%HRreserve, was 159 ± 12 bpm. Although SBP and MAP increased with interval exercise, DBP did not; these values were 161 ± 23, 94 ± 13, and 61 ± 13 mmHg, respectively at 32 min. RPE increased gradually with interval exercise, reaching 14 ± 2 at 32 min.

### 3.2 Venous vascular properties before and at 60 min after acute endurance exercise

**FIGURE 1:**
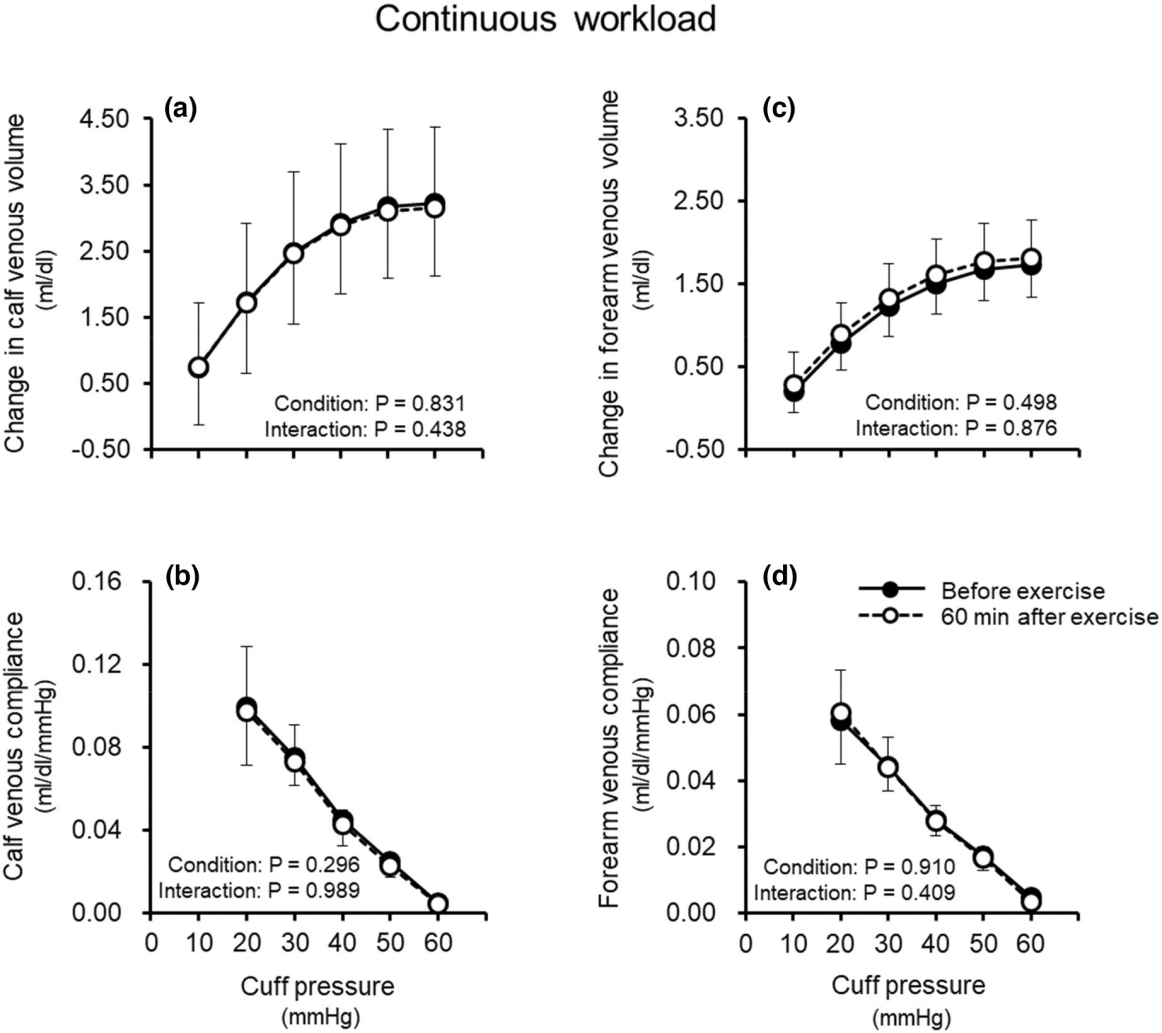
Effect of acute endurance exercise involving continuous workload on venous volume (a, c) and venous compliance (b, d) in the calf and forearm. Values are means ± standard deviation.

Figure 1 shows the effect of acute endurance exercise involving a continuous workload on venous volume and compliance in the calf and forearm. In both the calf and forearm, the cuff pressure–venous volume curves (Figure 1a,c) and the cuff pressure–venous compliance relationships (Figure 1b,d) did not differ between before and 60 min after acute endurance exercise involving a continuous workload.

**FIGURE 2:**
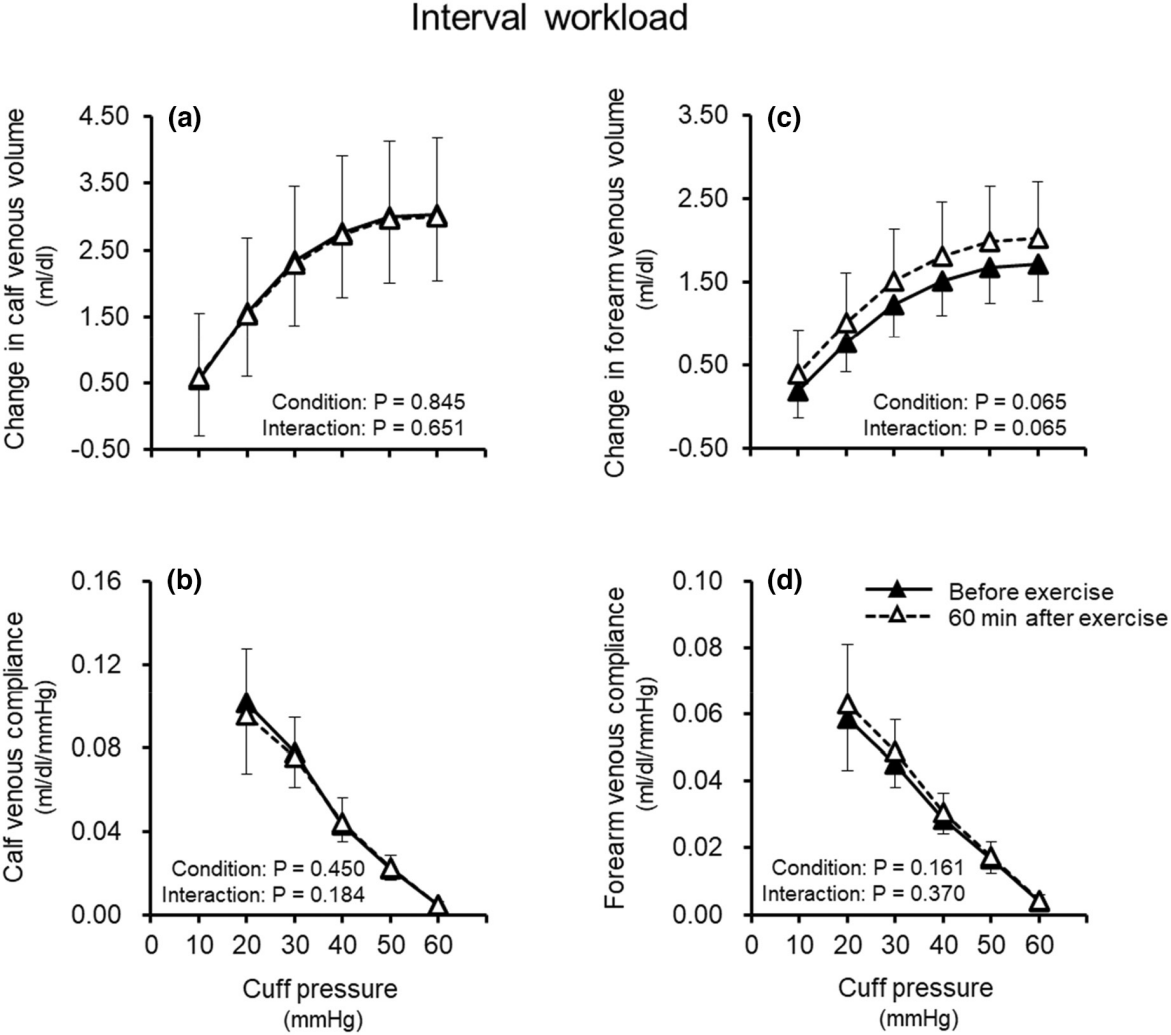
Effect of acute endurance exercise involving interval workload on venous volume (a, c) and venous compliance (b, d) in the calf and forearm. Values are means ± standard deviation.

Figure 2 shows the effect of acute endurance exercise involving an interval workload on venous volume and compliance in the calf and the forearm. In both the calf and forearm, changes in venous volume (Figure 2a,c) and venous compliance (Figure 2b,d) with cuff pressure did not differ between before and 60 min after acute endurance exercise involving an interval workload.

**FIGURE 3:**
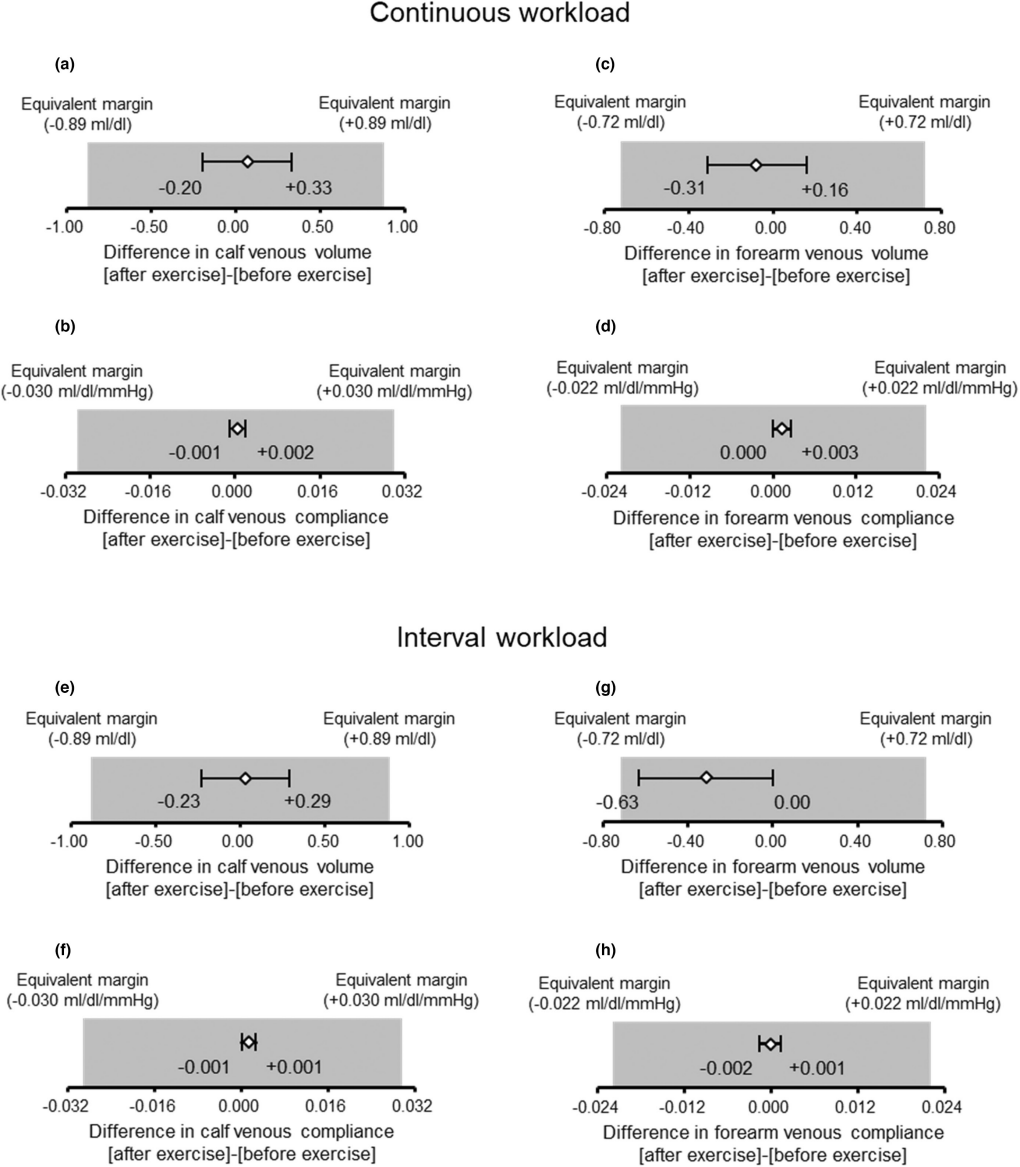
The 95% confidence interval for the difference between before and after acute exercise at continuous and interval workloads in venous volume and compliance in the limbs and the range of the predetermined equivalent margins. Continuous workload in venous volume (a, c) and venous compliance (b, d) in the calf and forearm. Interval workload in venous volume (e, g) and venous compliance (f, h) in the calf and forearm.

In addition, all 95%CI for the difference between before and after acute exercise at continuous and interval workloads in venous volume and compliance in the calf and the forearm were within the range of the predetermined equivalent margins (Figure 3a–h). These results indicated that the venous volume and compliance in the calf and the forearm before and after acute exercise were regarded as equivalence.

### 3.3 HR and BP before and at 60 min after acute endurance exercise

SBP (before exercise, 106 ± 10 mmHg; at 60 min after exercise, 106 ± 8 mmHg, *P* > 0.05) and MAP (before exercise, 75 ± 6 mmHg; at 60 min after exercise, 78 ± 5 mmHg, *P* > 0.05) were similar between before and 60 min after acute endurance exercise involving a continuous workload, whereas HR (before exercise, 59 ± 8 bpm; at 60 min after exercise, 65 ± 10 bpm, *P* < 0.05) and DBP (before exercise, 60 ± 6 mmHg; at 60 min after exercise, 63 ± 5 mmHg, *P* < 0.05) differed.

SBP (before exercise, 106 ± 10 mmHg; at 60 min after exercise, 106 ± 9 mmHg, *P* > 0.05) was similar between before and 60 min after acute endurance exercise involving an interval workload. In contrast, HR (before exercise, 59 ± 8 bpm; at 60 min after exercise, 67 ± 9 bpm, *P* < 0.05), DBP (before exercise, 60 ± 4 mmHg; at 60 min after exercise, 64 ± 5 mmHg, *P* < 0.05), and MAP (before exercise, 75 ± 6 mmHg; at 60 min after exercise, 78 ± 6 mmHg, *P* < 0.05) differed.

## 4 DISCUSSION

This study investigated the effect of acute endurance exercise on venous volume and compliance in the exercising limb (calf) and the non‐exercising limb (forearm). The primary finding is that venous volume and compliance in the calf and forearm did not change at 60 min after acute endurance exercise involving continuous and interval workloads. This result suggests that acute endurance exercise might not improve venous vascular properties in both the exercising and non‐exercising limbs.

Acute endurance exercise performed at both continuous and interval workloads did not change venous volume and compliance in the calf and forearm (Figures 1, 2, 3). To our knowledge, this is the first study to examine the effect of acute endurance exercise on venous vascular properties. However, some studies have investigated the effects of short‐term endurance exercise training on venous vascular properties. In contrast to our findings, those previous studies reported that short‐term endurance exercise training for 6–8 weeks caused increases in venous volume and compliance in the exercising limb (Iida et al., 2011; Oue et al., 2019) as well as an increase in venous volume in the non‐exercising limb (Miyachi et al., 2001; Oue et al., 2019). In addition, the degree of such venous vascular adaptation with short‐term exercise training for 8 weeks was greater for interval workload than continuous workload (Oue et al., 2019). From the previous findings on short‐term exercise training for 6–8 weeks and the present findings on acute exercise, it is likely that the effect of endurance exercise on venous vascular properties differs between short‐term exercise training for 6–8 weeks and acute exercise. It is assumed that a greater volume of exercise (intensity × duration × frequency) and thus repeating exercise stimuli (e.g., exercise training) at least for 6–8 weeks may be important to augment venous volume and compliance.

Although we do not know the exact reason why different frequencies of endurance exercise lead to different venous vascular properties, we consider the following possibility. Previous reports on arterial vascular adaptation have shown that short‐term exercise training for 8 weeks induces adaptation in both vascular functions (e.g., changes in the balance of sympathetic and parasympathetic nerve activity and/or increased release of vasodilator substances from the endothelium) and vascular structure (e.g., a change in the collagen‐to‐elastin ratio) (Birk et al., 2012; Brüel et al., 1998; Tinken et al., 2008), while acute exercise causes primarily changes in vascular function (Dawson et al., 2013; Tinken et al., 2009). These findings imply that the changes in function and structure in arterial vessels with endurance exercise might differ according to the exercise frequency (Green et al., 2017). If the same phenomenon occurs in the veins as in the arteries, the different changes in venous vascular function and structure with endurance exercise according to the exercise frequency might result in part from the different changes in venous volume and compliance between short‐term exercise training for 6–8 weeks and acute exercise. However, given that these possibilities are based on the findings of change in arterial vascular adaptation with endurance exercise, further studies are needed to identify the effect of endurance exercise on venous vascular function and structure.

Many studies have shown that arterial vascular health (e.g., endothelial function and vascular compliance) is improved by both short‐term exercise training for 4–8 weeks (Cameron & Dart, 1994; Kakiyama et al., 2005) and acute exercise (Kingwell et al., 1997; Tordi et al., 2010). However, from both previous studies and the present study, venous vascular properties seem to be increased by short‐term exercise training for 6–8 weeks (Iida et al., 2011; Miyachi et al., 2001; Oue et al., 2019) but are not likely to be changed by acute exercise (Figures 1, 2, 3). These findings suggest that the exercise volume (exercise intensity × duration × frequency) required to improve vascular health following endurance exercise may differ between the arteries and veins. Although we cannot explain the differences between the arteries and veins, it is speculated to be due to the differences in structure and/or function between them. Indeed, the number of endothelial cells as well as the elastin–collagen ratio differs between arteries and veins (Burton, 1954), and the sensitivity to NO and production of NO are higher in arteries than in veins (Fukaya & Ohhashi, 1996; Noris et al., 1995; Zhang et al., 2004; Zhang et al., 2006). Considering that veins play an important role in circulatory regulation (e.g., BP and cardiac output) (Rothe, 1983) and that elevated venous stiffness can be a factor in hypertension (Fink et al., 2000; Kooman et al., 1992; Oue et al., 2020; Overbeck, 1972; Safar & London, 1987; Simon, 1976; Takeshita & Mark, 1979), maintaining or increasing compliance of not only the arteries but also the veins might contribute to the prevention of cardiovascular disease. In addition, it has been reported that venous volume and compliance increased with endurance exercise training for 6–8 weeks (Iida et al., 2011, Miyachi et al., 2001, Oue et al., 2019), although it is unclear at what point in adaptation this occurs. Therefore, it is important to thoroughly investigate the intensity, duration, and frequency of endurance exercise programs that can induce increases in venous volume and venous compliance. This is the first study to show that acute exercise stimulus does not change venous volume and compliance in both the exercising and non‐exercising limbs, and that repeated endurance exercise (exercise training for at least 6–8 weeks) may be necessary to improve these venous vascular properties.

## 5 LIMITATIONS

This study has some limitations. First, calf volume measured by the VOP technique provides the vascular responses of all arteries, veins, and capillaries in the whole limb. Second, we used the venous collecting cuff pressure as a surrogate for intravenous pressure. As previously described (Monahan et al., 2001), we believe that this assumption is appropriate (Halliwill et al., 1999). Finally, this study involved young healthy participants and we cannot extrapolate our findings to older individuals or those with cardiovascular disease. Although these limitations do not directly affect the results of the present study, we need to thoroughly investigate the effect of endurance exercise on venous vascular function and structure in order to clarify the mechanisms underlying the adaptation of venous vascular properties with endurance exercise.

## 6 CONCLUSION

This study investigated the effect of acute endurance exercise involving continuous or interval workloads on venous volume and compliance in the calf (exercising limb) and in the forearm (non‐exercising limb). The primary finding was that venous volume and compliance in the calf and forearm did not change between before and 60 min after acute endurance exercise involving continuous or interval workloads. These results suggest that acute endurance exercise does not cause increases in venous volume and compliance in either the exercising or non‐exercising limbs, and that repeated endurance exercise (exercise training for at least 6–8 weeks) may be necessary to improve these venous vascular properties.

## AUTHOR CONTRIBUTIONS

Study design: Yasuhiro Iimura, Michiko Saito, and Anna Oue. Data acquisition: Yasuhiro Iimura and Anna Oue. Data analysis: Yasuhiro Iimura. Data interpretation and drafting of final manuscript: Yasuhiro Iimura, Michiko Saito, and Anna Oue. All authors reviewed and approved the final version of the manuscript.

## FUNDING INFORMATION

This research was supported by a Grant‐in‐Aid for Scientific Research (C) (18 K10974) from the Japan Society for the Promotion of Science.

## CONFLICT OF INTEREST

The authors have no financial conflicts of interest to declare.

## ETHICS STATEMENT

The purpose, procedures, and risks of the study were explained to the participants and their informed consent was obtained. This study was approved by the Human Ethics Committee of the Toyo University (approval no. 2015‐K‐02) and was conducted in accordance with the Declaration of Helsinki.
